# Clinical Effectiveness of Tislelizumab With Gemcitabine/Cisplatin Versus Gemcitabine/Cisplatin Alone as Adjuvant Therapy for High‐Risk Muscle‐Invasive Urothelial Carcinoma: A Real‐World Study

**DOI:** 10.1002/cam4.70661

**Published:** 2025-02-20

**Authors:** Yanjun Wang, Kaihua Zhong, Xingliang Tan, Qianghua Zhou, Lijuan Jiang, Kai Yao, Zhiming Wu

**Affiliations:** ^1^ Department of Urology Sun Yat‐Sen University Cancer Center Guangzhou China; ^2^ Department of Urology Meizhou People's Hospital Meizhou China

**Keywords:** cisplatin, disease‐free survival, gemcitabine, muscle‐invasive urothelial carcinoma, overall survival, tislelizumab

## Abstract

**Background:**

Muscle‐invasive urothelial carcinoma (MIUC) is a highly aggressive cancer associated with poor prognosis. Despite advancements in treatment, the optimal therapeutic approach remains unclear. Immune checkpoint inhibitors, when added to chemotherapy, have shown promise in improving patient outcomes.

**Aims:**

This study aimed to evaluate the efficacy and safety of adjuvant tislelizumab combined with gemcitabine/cisplatin (Tisle+GC) compared to GC alone in patients with high‐risk MIUC.

**Materials & Methods:**

We conducted a retrospective analysis of 117 patients with histologically confirmed pT3/4 and pN+ MIUC treated at our center between October 2016 and March 2023. Eligible patients received either Tisle+GC or GC alone, excluding those with prior neoadjuvant therapy. We compared disease‐free survival (DFS), overall survival (OS), and treatment‐related adverse events (AEs) between the two groups using Cox proportional hazards models and Kaplan–Meier estimates.

**Results:**

The Tisle+GC group showed significantly longer median DFS (19.08 vs. 9.06 months, HR = 0.114, *p* < 0.001) and OS (20.07 vs. 10.63 months, HR = 0.083, *p* = 0.026) compared to the GC group. Nerve tract invasion was identified as a significant predictor of poor outcomes (HR = 22.1, *p* = 0.003). Both groups experienced manageable grade 1–2 immune‐related AEs, with pruritus being the most common, followed by liver function abnormalities and thyroid disturbances. Nonhematologic toxicities in the Tisle+GC group included elevated aspartate aminotransferase and hyponatremia, while the GC group mainly reported vomiting. No treatment‐related fatalities occurred.

**Discussion:**

The addition of tislelizumab to GC chemotherapy significantly improved both DFS and OS in high‐risk MIUC patients. The safety profile was manageable, with immune‐related AEs being predictable and not life‐threatening. The findings support the potential of Tisle+GC as an effective adjuvant therapy.

**Conclusion:**

Tisle+GC is a promising adjuvant treatment for high‐risk MIUC, offering improved survival outcomes with a manageable safety profile. Further prospective studies are needed to confirm these results and establish the long‐term benefits of this combination therapy.

## 1 Introdution

Muscle‐invasive urothelial carcinoma (MIUC) is an aggressive cancer of the bladder that is highly invasive and responds poorly to current therapies [1, 2, 3]. After tumor cells have spread beyond the bladder wall, complete surgical resection is more difficult, and the survival rate is dramatically lower [4]. The current first‐line standard treatment for metastatic MIUC is cisplatin‐based combination chemotherapy [5, 6]. However, the response to this treatment is typically unsatisfactory, and most patients with MIUC have a poor prognosis [7]. The limitations of platinum‐based chemotherapy include the development of drug resistance, toxic side effects requiring dose reductions, and a lack of efficacy in patients whose tumors have intrinsic resistance to this therapy [8]. Although there is a significant need to improve the clinical outcomes of these patients, novel agents tested in combination with platinum compounds have only provided limited benefits. This is mainly because of the inability to predict individual responses or to tailor treatments on the basis of tumor biology. Thus, more effective strategies are urgently needed to overcome drug resistance and improve survival in patients with advanced MIUC [9].

Recent advancements in cancer immunotherapy have provided new opportunities for the management of MIUC [10, 11]. For example, inhibitors of ***programed death protein 1 (PD1) and programed death ligand 1 have emerged as potentially valuable therapeutic agents [12, 13, 14] because they block immune checkpoints and enhance the body's immune response to cancer cells. The CheckMate 274 trial was a landmark study that broadened our understanding of the possible role of adjuvant immunotherapy for the treatment of patients with high‐risk MIUC [15]. This trial showed that nivolumab (NIVO), a PD1 inhibitor, significantly improved disease‐free survival (DFS) following radical surgery in patients with MIUC, thus supporting the inclusion of adjuvant immunotherapy in standard treatment protocols for these patients. Similarly, the CheckMate 901 trial compared gemcitabine/cisplatin (GC) alone and NIVO combined with GC as a front‐line treatment for metastatic urothelial carcinoma. The results showed that the addition of NIVO led to significant improvements in overall survival (OS) and progression‐free survival [16].

Despite the success of these and other studies that examined the effect of a PD‐1 inhibitor in combination with chemotherapy for advanced urothelial carcinoma, several important gaps remain [16, 17, 18]. First, the two PD‐1 inhibitors used in previous trials (NOVO and tislelizumab) are not yet widely available in China and some other regions, so clinical data from patients in East Asia are still limited. Differences in the genetic backgrounds and environmental exposures of East Asian patients suggest that regional studies are warranted [19]. Furthermore, no studies have yet directly compared the efficacy of PD‐1 inhibitor‐based combinations with standard adjuvant GC regimens in patients with MIUC.

The present study addressed these two limitations by retrospectively investigating the effect of tislelizumab combined with a standard GC regimen in Chinese patients with metastatic MIUC. When tislelizumab becomes more available locally, our results could help guide the development of novel first‐line treatments and improve outcomes for this high‐risk group of patients in our region [20]. We therefore compared standard adjuvant GC chemotherapy alone and the combination of tislelizumab with standard GC chemotherapy on the DFS and OS of patients with advanced MIUC. Our comparison of patients in these two groups may provide important information regarding immunotherapeutic approaches for the treatment of MIUC in patients from Asia.

## 2 Methods

### 2.1 Eligibility

We retrospectively reviewed the records of high‐risk MIUC patients (*n* = 117), who were originally from multiple medical centers and received treatment at the Sun Yat‐sen University Cancer Center (Guangzhou, China) between October 2016 and March 2023. Eligible participants were adults (age ≥ 18 years) with histologically confirmed pT3/4 and pN+ UC. Patients who received prior neoadjuvant therapy were excluded. Excluding these patients strengthens the study by ensuring a more uniform population, reducing potential confounding effects from pretreatment, and allowing for a clearer evaluation of the outcomes specific to the combination therapy being studied. This study was approved by the Ethics Committee of Sun Yat‐sen University Cancer Center (approval no.: SL‐G2023‐230‐01) and was conducted according to the guidelines of the Declaration of Helsinki. Because of the retrospective and anonymous nature of this study, informed consent was not required from the patients.

### 2.2 Trial Design and Treatments

**FIGURE 1:**
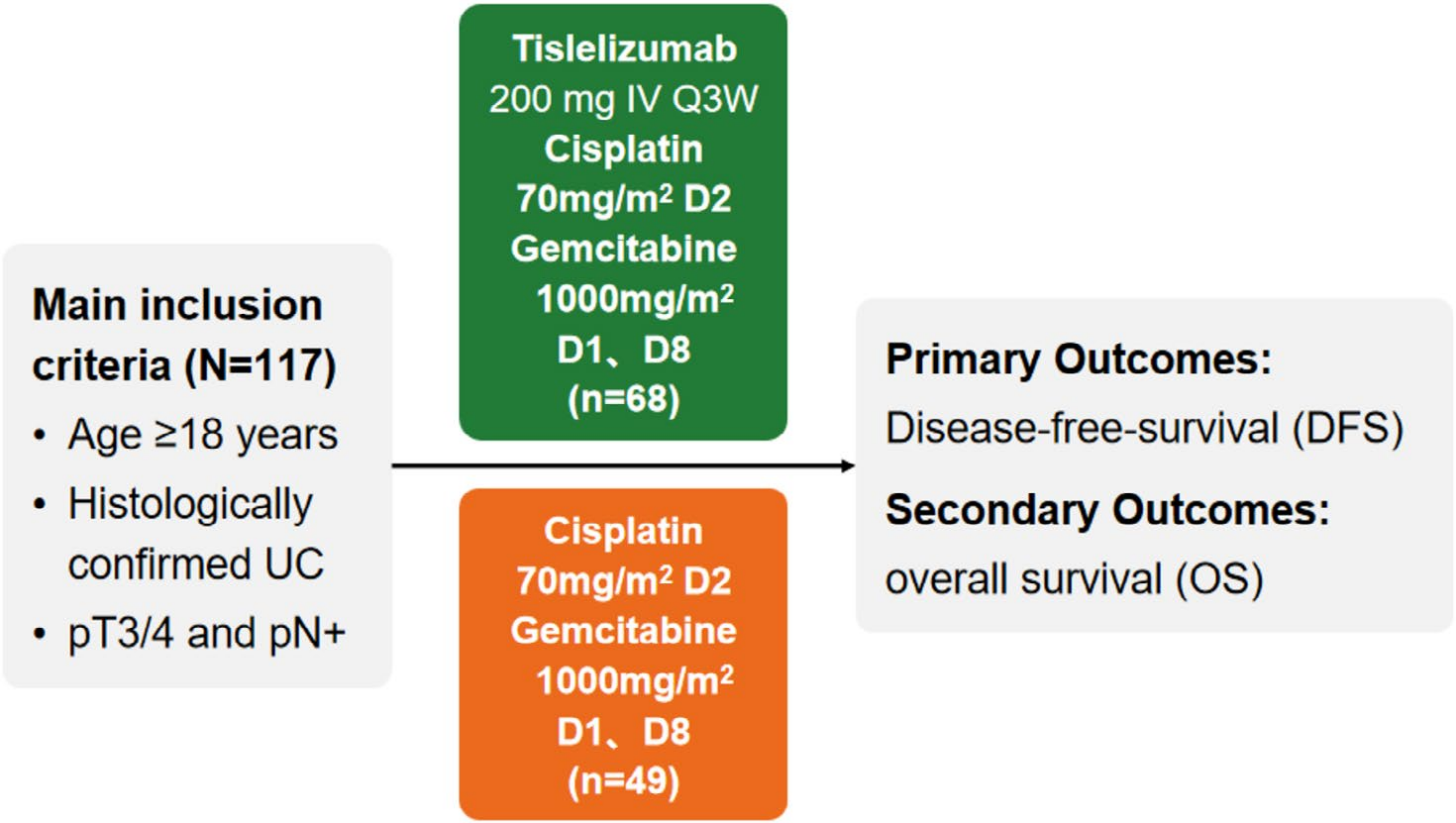
Overview of study design and treatment regimens for patients with muscle‐invasive urothelial carcinoma.

Upon the completion of radical surgery, participants were allocated to one of the two treatment groups: a combination of tislelizumab with GC (Tisle+GC) or GC alone. The Tisle+GC group (*n* = 61) received 2 to 6 cycles of cisplatin (70 mg/m^2^ on day 1) and gemcitabine (1000 mg/m^2^ on days 1 and 8) every three weeks, followed by 2 to 17 cycles of tislelizumab (200 mg every 3 weeks). The GC group (*n* = 75) received the same GC regimen, but no tislelizumab. The primary endpoint was DFS, and the secondary endpoints were OS and safety (Figure 1). The mean follow‐up durations were 15.17 months for DFS and 16.77 months for OS. The Tisle+GC group underwent a median of three chemotherapy cycles (range: 1–6; IQR: 1–3) and three immunotherapy cycles (range: 1–17; IQR: 2–3), whereas the GC group had a median of three chemotherapy cycles (range: 1–6; IQR: 2–3). Ten patients were lost to follow‐up, including six in the GC group and four in the Tisle+GC group.

### 2.3 Statistical Analysis

Several statistical methods were used to evaluate the effect of the different treatments on outcomes. The differences in DFS and OS between the two groups were analyzed using the Kaplan–Meier method with the log‐rank test to determine the statistical significance of differences. Moreover, hazard ratios (HRs) were calculated using univariate and multivariate Cox proportional hazards regression models, with the multivariate model adjusting for multiple potential confounders. The statistical significance level was set at *p* < 0.05, and all analyses were performed using an appropriate statistical software package. The 95% confidence intervals (CIs) were calculated to provide a range of plausible values for the effect size on the basis of the data. All statistical analyses were performed using SPSS 22.0 and Excel.

## 3 Results

### 3.1 Patient Characteristics

A comparison of the baseline characteristics of patients in the Tisle+GC and GC groups (Table 1) showed that the two groups were well balanced in terms of age (median: 64.29 vs. 64.12 years, *p* = 0.866), sex (male: 79.41% vs. 69.39%, *p* = 0.215), initial tumor origin (bladder vs. upper urinary tract, *p* = 0.885), histological type (UC vs. histologic variant UC, *p* = 0.366), pathological conditions (intravascular cancer embolus vs. none, *p* = 0.124; nerve tract invasion vs. none, *p* = 0.467), postoperative lymph node status (N+ vs. N−, *p* = 0.717), and pathological stage (pT2 vs. pT3‐4, *p* = 0.102).

**TABLE 1:** Baseline characteristics of the two groups.^a^

Characteristic	Tisle+GC (*N* = 68)	GC (*N* = 49)	*P*
**Age, years**	64.29 (35–85)	64.12 (34–84)	0.866
**Male sex**	54 (79.41%)	34 (69.39%)	0.215
**Initial tumor origin**			0.885
Bladder	*n* (37%)	*n* (26%)	
Upper urinary tract (renal pelvis/ureter)	*n* (31%)	*n* (23%)	
**Histological type**			0.366
UC	*n* (56%)	*n* (37%)	
Histologic variant UC	*n* (12%)	*n* (12%)	
**Pathologic condition**			0.124
Intravascular cancer embolus	*n* (25%)	*n* (25%)	
None	*n* (43%)	*n* (24%)	
**Nerve tract invasion**			0.467
Yes	*n* (18%)	*n* (16%)	
No	*n* (50%)	*n* (33%)	
**Postoperative lymph node status**			0.717
N+	*n* (37%)	*n* (24%)	
N−	*n* (31%)	*n* (25%)	
**Postoperative pathologic stage**			0.102
pT2	*n* (20%)	*n* (8%)	
pT3–4	*n* (48%)	*n* (41%)	

^a^ Data are presented as median (range) or *n* (%).

### 3.2 Disease‐Free Survival

**FIGURE 2:**
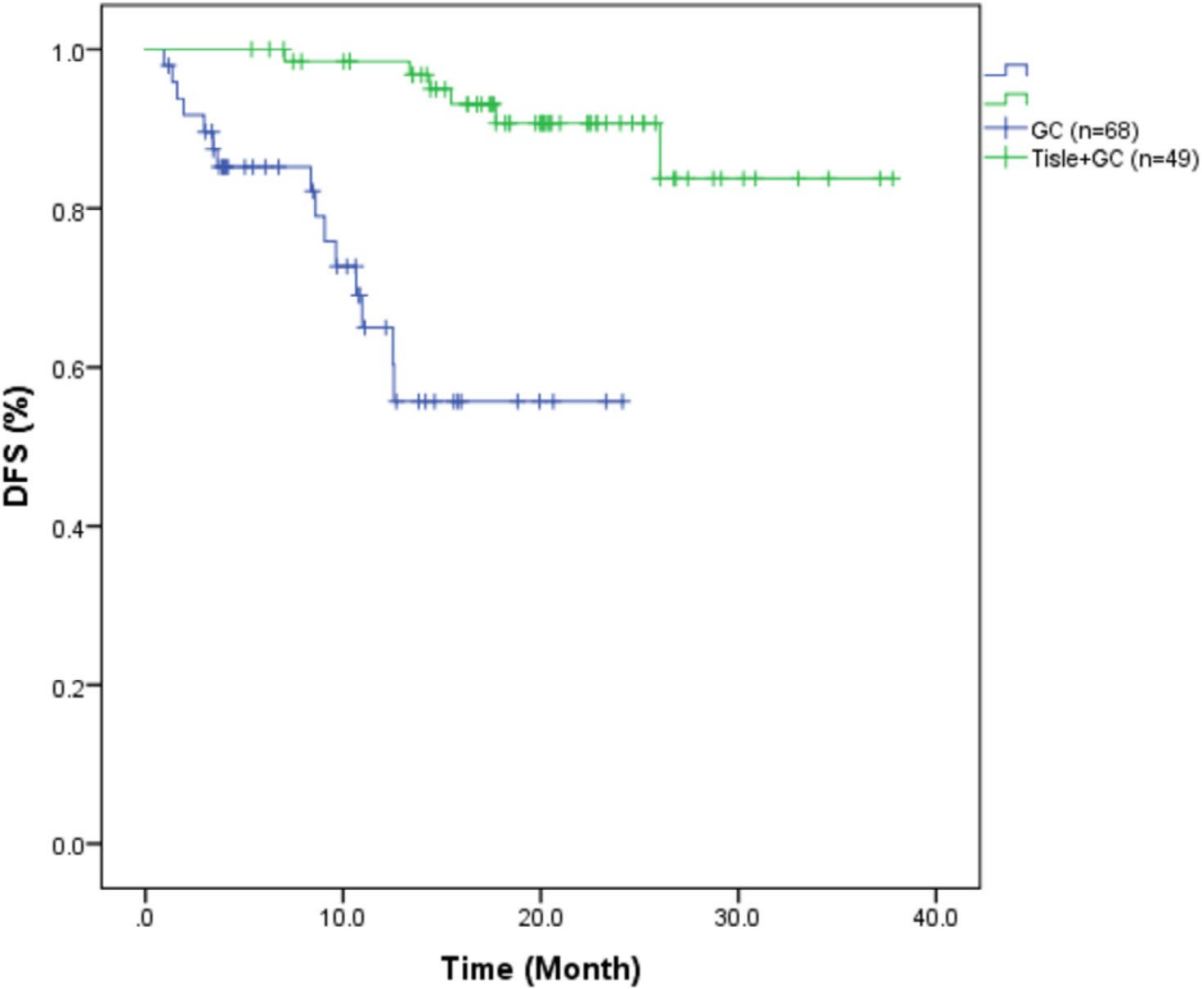
Kaplan–Meier curves for disease‐free survival in patients with muscle‐invasive urothelial carcinoma who received tislelizumab with gemcitabine and cisplatin (Tisle+GC) or gemcitabine and cisplatin alone (GC).

The median DFS was 19.08 months (95% CI: 6.02–37.34) in the Tisle+GC group and 9.06 months (95% CI: 0.99–23.93) in the GC group (Figure 2). The corresponding HR for DFS was 0.114 (95% CI: 0.040–0.324), indicating a significantly lower risk of recurrence or metastasis in the Tisle+GC group (*p* < 0.001).

### 3.3 Overall Survival

**FIGURE 3:**
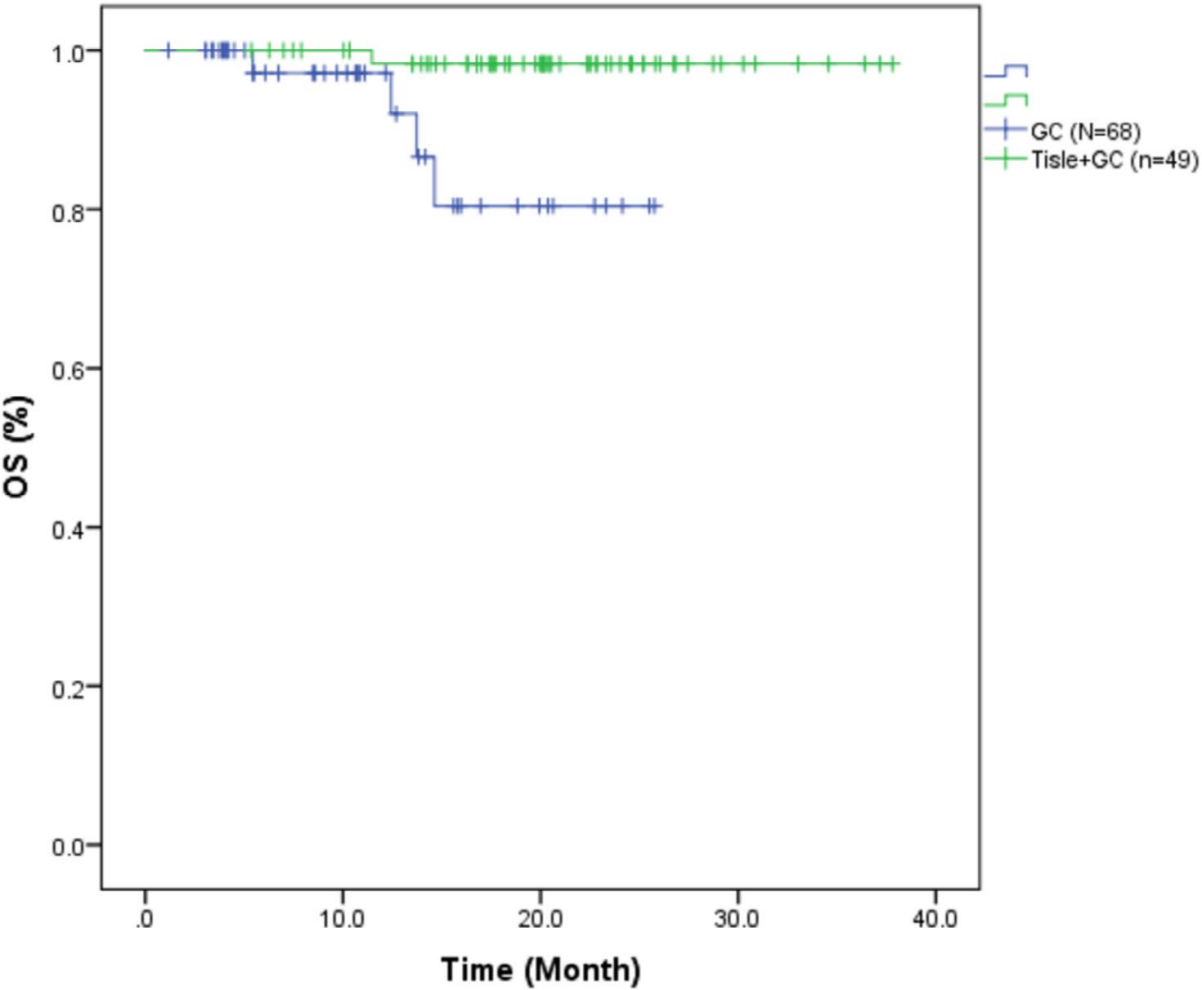
Kaplan–Meier curve for overall survival in patients with muscle‐invasive urothelial carcinoma who received tislelizumab with gemcitabine and cisplatin (Tisle+GC) or gemcitabine and cisplatin alone (GC).

The median OS was 20.07 months (95% CI: 6.02–37.34) in the Tisle +GC group and 10.63 months (95% CI: 1.63–25.70) in the GC group (Figure 3). The HR for OS was 0.083 (95% CI: 0.009–0.747), indicating there was also a significantly lower risk of death in the Tisle+GC group (*p* = 0.026).

### 3.4 Bivariate Analysis

We performed a bivariate Cox regression analysis to identify potential predictors of DFS (Table 2). Among the nine examined clinical characteristics, intravascular cancer embolus (HR = 0.206, 95% CI: 0.075–0.564, *p* = 0.002), nerve tract invasion (HR = 0.387, 95% CI: 0.164–0.914, *p* = 0.030), and receipt of Tisle+GC (HR = 0.114, 95% CI: 0.040–0.324, *p* < 0.001) were significantly associated with a longer DFS. Patient age, sex, initial tumor origin, lymph node status, histological type, and pathologic stage had no significant relationship with DFS (all *p* > 0.05).

**TABLE 2:** Bivariate Cox regression analysis of factors associated with disease‐free survival.

Characteristic	HR (95% CI)	*P*
Age: < 60 vs. ≥ 60 years	1.316 (0.482–3.597)	0.592
Sex: male vs. female	0.512 (0.151–1.737)	0.282
Initial tumor origin: bladder vs. upper urinary tract (renal plevis/ureter)	0.647 (0.268–1.563)	0.333
Lymph node status: N−(0) vs. N+ (1–3)	1.436 (0.594–3.468)	0.421
Histological type: UC vs. histologic variant UC	1.603 (0.584–4.398)	0.359
Intravascular cancer embolus vs. none	0.206 (0.075–0.564)	**0.002**
Nerve tract invasion vs. none	0.387 (0.164–0.914)	**0.030**
Treatment: Tisle+GC vs. GC	0.114 (0.040–0.324)	**0.000**
Pathologic stage: pT2 vs. pT3‐4	0.734 (0.296–1.823)	0.506

*Note:* bold *P*‐values correspond to subgroups that require special attention.

Abbreviation: Ref, reference group.

### 3.5 Multivariate Analysis

We then performed multivariate Cox regression analysis with control for multiple confounding factors (age, sex, initial tumor origin, lymph node status, histological type, nerve tract invasion, and pathologic stage; Table 3). The results showed that only intravascular cancer embolus (HR = 0.290, 95% CI: 0.092–0.906, *p* = 0.033) and receipt of Tisle+GC therapy (HR = 0.097, 95% CI: 0.031–0.305, *p* < 0.001) were significantly associated with a longer DFS.

**TABLE 3:** Multivariate Cox regression of characteristics associated with disease‐free survival.

Characteristic	HR (95% CI)	*P*
Age: < 60 vs. ≥ 60 years	1.1179 (0.364–3.811)	0.784
Sex: male vs. Female	0.535 (0.142–2.013)	0.355
Initial tumor origin: bladder vs. upper urinary tract (renal plevis/ureter)	0.713 (0.233–2.177)	0.552
Lymph node status: N–(0) vs. N+ (1–3)	1.291 (0.401–4.156)	0.669
Histological type: UC vs. histologic variant UC	1.791 (0.516–6.221)	0.359
Intravascular cancer embolus vs. none	0.290 (0.092–0.906)	**0.033**
Nerve tract invasion vs. none	0.654 (0.229–1.870)	0.428
Treatment: Tisle+GC vs. GC	0.097 (0.031–0.305)	**0.000**
Pathologic stage: pT2 vs. pT3–pT4	0.710 (0.219–2.298)	0.568

*Note:* The bold *P*‐values correspond to subgroups that require special attention.

Abbreviation: Ref, reference group.

### 3.6 Safety

**FIGURE 4:**
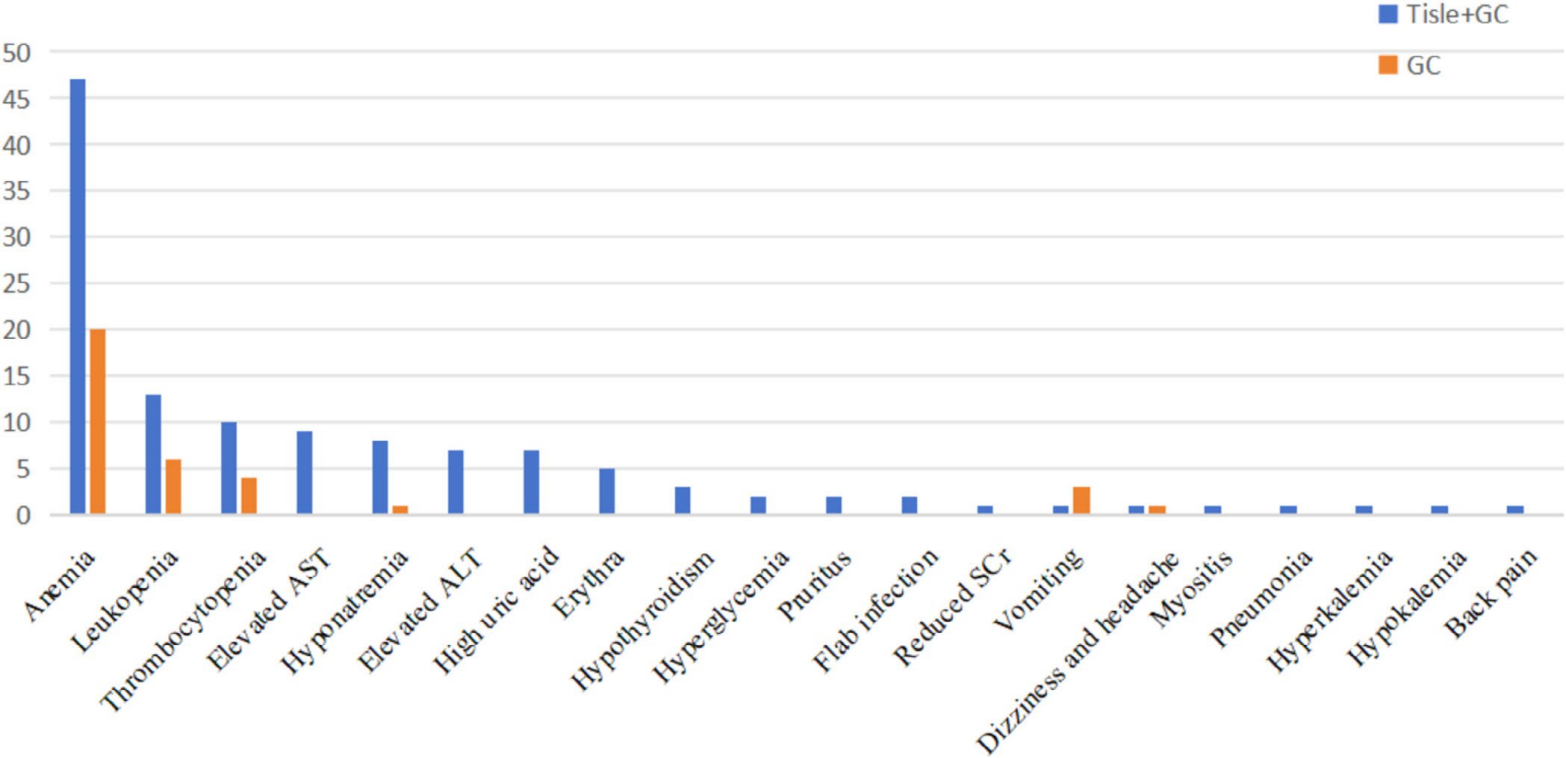
Adverse events of all grades in the two treatment groups.

We also recorded adverse events (AEs) in the two treatment groups (Table 4 and Figure 4). In the Tisle+GC group, the most common hematologic toxicities were anemia (69.12% for all grades, 4.41% for grade ≥ 3), leukopenia (19.12% for all grades, 4.41% for grade ≥ 3), and thrombocytopenia (14.71% for all grades, 5.88% for grade ≥ 3). In the GC group, anemia was also the most common hematologic toxicity (40.82% for all grades, 8.16% for grade ≥ 3), followed by leukopenia (12.24% for all grades, 6.12% for grade ≥ 3) and thrombocytopenia (8.16% for all grades, 2.04% for grade ≥ 3).

**TABLE 4:** Adverse events in the two treatment groups.

	Tisle+GC (*N* = 68)		GC (*N* = 49)	
Adverse event	All grades *n* (%)	Grade ≥ 3 *n* (%)	All grades *n* (%)	Grade ≥ 3 *n* (%)
**Hematologic toxicity**				
Anemia	47 (69.12)	3 (4.41)	20 (40.82)	4 (8.16)
Leukopenia	13 (19.12)	3 (4.41)	6 (12.24)	3 (6.12)
Thrombocytopenia	10 (14.71)	4 (5.88)	4 (8.16)	1 (2.04)
**Other events**				
Elevated AST	9 (13.24)	1 (1.47)		
Hyponatremia	8 (11.76)	0	1 (2.04)	0
Elevated ALT	7 (10.29)	0		
High uric acid	7 (10.29)	0		
Erythra	5 (7.35)	0		
Hypothyroidism	3 (4.41)	0		
Hyperglycemia	2 (2.94)	0		
Pruritus	2 (2.94)	0		
Flab infection	2 (2.94)	0		
Reduced SCr	1 (1.47)	0		
Vomiting	1 (1.47)	0	3 (6.12)	0
Dizziness and headache	1 (1.47)	0	1 (2.04)	0
Myositis	1 (1.47)	0		
Pneumonia	1 (1.47)	0		
Hyperkalemia	1 (1.47)	0		
Hypokalemia	1 (1.47)	0		
Back pain	1 (1.47)	0		

The other AEs were diverse and generally rare (Table 4). In the Tisle+GC group, the most common nonhematologic AEs were elevated aspartate amino transferase (AST; 13.24% for all grades, 1.47% for grade ≥ 3), hyponatremia (11.76% for all grades), elevated alanine amino transferase (ALT; 10.29% for all grades), and high uric acid (10.29% for all grades). In the GC group, vomiting (6.12% for all grades) and dizziness and headache (2.04% for all grades) were the most frequent nonhematologic AEs.

## 4 Discussion

Our retrospective real‐world study offers valuable insights regarding the relative efficacy of Tisle+GC versus GC alone for the treatment of MIUC. The two groups were not significantly different in terms of age, sex distribution, or tumor origin (bladder vs. upper urinary tract). Although the two groups had many similar characteristics, the DFS was significantly better in the Tisle+GC group. This finding suggested that the addition of tislelizumab to the standard GC regimen prolonged DFS in patients with MIUC, a finding that warrants further exploration in larger, randomized controlled trials. In fact, the much longer median DFS time in the Tisle+GC group (19.08 vs. 9.06 months, *p* < 0.001) strongly suggests that the addition of tislelizumab to the traditional GC regimen offers a very significant benefit. Similarly, our Kaplan–Meier analysis also demonstrated a significantly longer median OS in the Tisle+GC group (20.07 months vs. 10.63 months, *p* = 0.026).

The CheckMate 274 trial, an influential study that shaped current guidelines, demonstrated the efficacy of the adjuvant NIVO (a PD1 inhibitor) in patients with high‐risk MIUC who received radical surgery [15]. The positive results from this trial led the National Comprehensive Cancer Network and European Association of Urology to adopt guidelines for adjuvant immunotherapy for this cohort of patients [21]. Our therapeutic strategy of adjuvant therapy of tislelizumab with GC chemotherapy appeared even more effective than the strategy recommended in these guidelines. This assertion is also supported by recent findings from the CheckMate 901 study [16], which concluded that first‐line treatment consisting of an immune checkpoint inhibitor with GC chemotherapy provided a survival advantage for patients with advanced urothelial carcinoma. The pivotal CheckMate 901 study underpins the rationale for our approach, and taken together with our results suggests that the translation of this combination therapy may be effective against earlier stages of localized or locally advanced MIUC. By extrapolating the principles from the CheckMate 901 study, our current research supports a “treatment intensification” hypothesis, which posits that the synergistic effects of two treatment modalities could lead to improved OS, similar to what occurs in the metastatic setting. The early use of this intensified regimen for treatment of localized cancers might lead to improved patient outcomes and align with the emerging trend of utilizing systemic therapies earlier during the course of disease to better leverage their potential benefits.

Our univariate analysis showed that nerve tract invasion significantly increased the risk of death in patients with MIUC. Similarly, our observed trend of increased risk of mortality in patients with intravascular cancer embolus underscores the adverse prognostic implications of vascular involvement. The significantly lower risk of mortality associated with Tisle+GC relative to GC alone provides strong support for the addition of tislelizumab to the standard GC regimen. This finding reflects the potential therapeutic benefits of tislelizumab and also emphasizes the critical role of treatment choice on survival outcomes. Future research should continue to investigate these and other possible factors to further refine treatment strategies and improve survival outcomes in patients with MIUC.

Overall, our Tisle+GC group had a greater incidence of AEs than the GC group, particularly hematologic toxicities and the spectrum of nonhematologic events. However, most of the observed AEs were low grade, and only a small proportion of patients from both groups experienced grade 3 or higher toxicities. These findings align with the incidence of AEs in previous studies of immune checkpoint inhibitors [22, 23, 24]. The most common AE in our study—pruritus—although manageable and not life‐threatening, can significantly impact patient quality of life [25]. Strategies to predict, prevent, and manage pruritus could greatly enhance patient comfort. The presence of liver function abnormalities in some patients underscores the potential hepatotoxic effects of tislelizumab and reinforces the importance of regular monitoring of liver enzymes [26, 27]. Similarly, the presence of thyroid function abnormalities in the Tisle+GC group suggests that this treatment may impact the regulation of thyroid hormone [28]. Clinicians should be aware of these potential adverse effects and should consider routine testing of thyroid function. The incidence of hyperglycemia [29], albeit rare in our study, suggests that Tisle+GC may impact glucose metabolism, a finding that may have implications for patients with preexisting diabetes or a high risk for developing diabetes. Our documentation of AEs highlights the importance of regular monitoring and prompt management of AEs to ensure optimal patient comfort and outcomes and can be used to guide future research that investigates strategies to minimize these side effects and improve the safety profile of Tisle+GC treatment.

Despite the promising findings of our study, certain limitations must be acknowledged. First, the retrospective nature of our investigation introduced the potential for selection bias, because the treatment allocations were not randomized. This could have led to an imbalance of confounding variables that could potentially influence the outcomes. Additionally, our sample size was relatively small, limiting the statistical power to detect differences, especially in subgroup analyses. Our single‐center study design also limits the generalizability of the results, because our patient population might not fully represent the broader demographic population affected by MIUC in China. Furthermore, the follow‐up period, while adequate to observe initial treatment responses, may not have been long enough to fully assess some long‐term outcomes, such as OS and late‐onset AEs. Future multicenter and randomized controlled trials with larger sample sizes and longer follow‐up durations are necessary to validate our findings and to optimize clinical practice guidelines.

## 5 Conclusions

In conclusion, our data provide compelling evidence for the real‐world effectiveness of Tisle+GC as an adjuvant therapy for patients with MIUC who present with pT3/4 disease or positive nodal status (pN+). These findings suggest that this combination therapy may be a valuable treatment option for patients with features of high‐risk MIUC. The benefit of including tislelizumab in standard GC regimens highlights the potential value of using immune checkpoint inhibitors during adjuvant therapy. Furthermore, the favorable safety profile, with manageable immune‐related AEs, underscores the value of this regimen in clinical practice. Clinicians must monitor and manage these AEs effectively to maintain a balance between treatment efficacy and patient quality of life. These results warrant further investigation and validation in larger prospective trials to confirm the benefits and safety of Tisle+GC as an adjuvant treatment for MIUC. The promising outcomes from this study could pave the way for new standards in the care of patients with advanced urothelial carcinoma.

## Author Contributions

K.Y. and Z.W. conceived and designed the research; Q.Z. and L.J. collected data and conducted research; Q.Z. and L.J. analyzed and interpreted data; Y.W. wrote the initial draft; X.T. revised the manuscript; K.Y. and Z.W. had primary responsibility for the final content. All authors read and approved the final version of the manuscript.

## Ethics Statement

This study was approved by the Ethics Committee of Sun Yat‐sen University Cancer Center (approval no.: SL‐G2023‐230‐01) and was conducted according to the guidelines of the Declaration of Helsinki. Because of the retrospective and anonymous nature of this study, informed consent was not required from the patients.

## Conflicts of Interest

The authors declare no conflicts of interest.

## Data Availability

The datasets generated and analyzed during the current study are not publicly available because of limitations of ethical approval involving patient data and anonymity but are available from the corresponding author on reasonable request.
